# Enhanced bioactive properties of Biodentine^TM^ modified with bioactive glass nanoparticles

**DOI:** 10.1590/1678-77572016-0209

**Published:** 2017

**Authors:** Camila CORRAL NUÑEZ, Cristian COVARRUBIAS, Eduardo FERNANDEZ, Osmir Batista de OLIVEIRA

**Affiliations:** 1 Universidad de Chile, Facultad de Odontología, Departamento de Odontología Restauradora, Santiago, Chile; 2 Universidade Estadual Paulista - UNESP, Faculdade de Odontologia, Departmento de Odontologia Restauradora, Araraquara, Brazil.; 3 Universidad de Chile, Facultad de Odontología, Instituto de Investigación en Ciencias Odontológicas, Laboratorio de Nanobiomateriales, Santiago, Chile.

**Keywords:** Apatite-forming ability, Bioactive glass, Bioactivity, Biodentine, Nanocomposites

## Abstract

**Objective:**

To prepare nanocomposite cements based on the incorporation of bioactive glass nanoparticles (nBGs) into Biodentine^TM^ (BD, Septodent, Saint-Maur-des-Fosses Cedex, France) and to assess their bioactive properties.

**Material and Methods:**

nBGs were synthesised by the sol-gel method. BD nanocomposites (nBG/BD) were prepared with 1 and 2% nBGs by weight; unmodified BD and GC Fuji IX (GIC, GC Corporation, Tokyo, Japan) were used as references. The *in vitro* ability of the materials to induce apatite formation was assessed in SBF by X-ray diffraction (XRD), attenuated total reflectance with Fourier transform infrared spectroscopy (ATR-FTIR), and scanning electron microscopy (SEM) with energy dispersive X-ray (EDX) analysis. BD and nBG/BD were also applied to dentine discs for seven days; the morphology and elemental composition of the dentine-cement interface were analysed using SEM-EDX.

**Results:**

One and two percent nBG/BD composites accelerated apatite formation on the disc surface after short-term immersion in SBF. Apatite was detected on the nBG/BD nanocomposites after three days, compared with seven days for unmodified BD. No apatite formation was detected on the GIC surface. nBG/BD formed a wider interfacial area with dentine than BD, showing blockage of dentine tubules and Si incorporation, suggesting intratubular precipitation.

**Conclusions:**

The incorporation of nBGs into BD improves its *in vitro* bioactivity, accelerating the formation of a crystalline apatite layer on its surface after immersion in SBF. Compared with unmodified BD, nBG/BD showed a wider interfacial area with greater Si incorporation and intratubular precipitation of deposits when immersed in SBF.

## Introduction

Biodentine^TM^ (BD), a tricalcium silicate-based cement, was developed as a dentine substitute with clinical applications, including direct and indirect pulp capping^9^ and the restoration of coronal dentine^16^. For some of these applications, the material may come into direct contact with pulpal tissues or with deeply carious dentine, making its biocompatibility and ability to seal in moist environments relevant clinical properties. It is well established that the placement of a permanent, properly sealed restoration is crucial to clinical success in indirect and direct pulp therapies^11^, a property that closely relates to the bioactivity of the applied restorative material.

Bioactivity is defined as the capacity of a material to “elicit a specific biological response at the interface of the material which results in the formation of a bond between the tissues and the material”^2^. BD has been shown *in vitro* to induce the formation of calcium and phosphorous surface precipitates after immersion in biological fluids^7^ and allows the formation of an interfacial layer with dentine^8^ ^,^ ^13^. The mechanism of action proposed for BD effect on dentin is that, first, a degradation of collagenous components occurs due to an alkaline caustic effect, which forms a porous structure that facilitates the permeation of Ca^2+^, OH^-^, and CO_3_^2-^ions, mineralising this substrate^26^. *In vivo* studies demonstrated the formation of reparative dentine after BD pulp capping, which is an evidence for its bioactivity, resulting in a bond with the tissue^19^; however, there are concerns about the stability of this interfacial layer, since only amorphous-calcium-phosphate has been identified, not dentine-like hydroxyapatite^13^.

Bioactive glass (BG) is a well-known bioactive ceramic material that has gained attention due to its ability to chemically bond with hard tissues through the formation of an apatite layer on its surface^12^. This apatite layer forms following solution-mediated dissolution of the glass^12^. It has been proposed that when BG is in contact with physiological fluids a series of reactions occurs, including: a rapid ionic exchange, creation of silanol bonds on the glass surface, increase of pH with formation of silica-rich region, migration of Ca^+2^ and PO_4_^3-^ groups from the solution, which leads to the formation of an apatite layer^12^. For this ability, it has been incorporated into a range of products, including synthetic bone grafts to induce hard-tissue regeneration and toothpastes that treat hypersensitivity^12^. Recently, a study evaluated the use of BG as a dentine substitute; however, the BG particle size was ca. 700 μm, resulting in poor cavity adaptation with empty spaces between BG particles, and therefore the use of smaller particles was recommended^6^.

Contemporary manufacturing processes allow the synthesis of nanometre-size BG, and BG nanoparticles (nBGs) have superior bioactivity compared with traditional micrometre-size BG, accelerating the formation of hydroxyapatite when it is incorporated into different biomaterials^23^. Brushing exposed dentin tubules with nBGs forms occluding and tightly bonded hydroxyapatite rods that extend deep into dentinal tubules^4^. Hydroxyapatite is less susceptible to degradation than other amorphous calcium phosphate phases with different Ca/P ratio than the stoichiometric crystalline hydroxyapatite (1.67)^25^. Therefore, it is expected that a hydroxyapatite-based interface provides a more stable seal than an amorphous-calcium-phosphate-based interface, as seen in BD/dentine interface^13^. To the best of our knowledge, nBGs have not been utilised in the formulation or modification of calcium silicate-based cements. The incorporation of nBGs into BD could enhance BD’s bioactive properties to stimulate the formation of crystalline hydroxyapatite, equivalent to that of dentine hard tissue.

The aim of this study was to prepare BD modified with nBGs and assess the *in vitro* and *ex vivo* bioactivity of these novel nanocomposite cements (nBG/BDs). The hypothesis is that the incorporation of nBG into BD improves its bioactivity, inducing a higher degree of mineralisation in dentin-cement interface.

## Material and methods

### Material

This study included two commercially available cements, Biodentine (BD, Septodent, Saint-Maur-des-Fosses Cedex, France; Lot No. B08571) and GC Fuji IX Capsule (GIC, GC Corporation, Tokyo, Japan; Lot No. 1208061), and two experimental nanocomposite cements (nBG/BD), 1%nBG/BD and 2%nBG/BD, which were composed of BD with 1% nBGs and 2% nBGs by weight respectively.

### Preparation of nanocomposite cements.

nBG particles (size ca. 40-70 nm) were synthesised by the sol-gel method, using the following previously-described molar composition: 58SiO_2_:40CaO:5P_2_O_5_ ^23^. 1%nBG/BD and 2%nBG/BD were prepared by adding 7 and 14 mg of nBG powder to the BD capsule, respectively. The resulting nBG/BD nanocomposites powder was then mixed dry within the BD capsule in an amalgamator (Ultramat 2, SDI, Australia) for 30 seconds. Five drops of BD liquid were then added to the capsule before mixing, according to the BD manufacturer’s instructions.

### *In vitro* bioactivity assay

The ability of the cement materials to induce the formation of apatite was assessed in acellular SBF, which was prepared as described by Kokubo, et al.^15^ (1990) using the standard ion composition (Na^+^ 142.0, K^+^ 5.0, Mg^2+^ 1.5, Ca^2+^ 2.5, Cl^-^ 147.8, HCO_3_^-^ 4.2, HPO_4_^2-^ 1.0, SO_4_^2-^ 0.5 mM) and buffered at pH 7.4 at 37°C. For this purpose, discs of material, measuring 7 mm in diameter and 2.5 mm thick, were prepared (for BD and GIC, the manufacturers’ mixing instructions were followed) and allowed to fully set during incubation at 37°C and 100% humidity for 24 hours. Specimens were individually immersed in 50 mL of SBF, using polyethylene containers, for three or seven days at 37°C in a thermostatic bath.

### Materials and surface deposit characterisation

Structures of the set cement materials and surface deposits formed in the SBF assays were examined by X-ray diffraction analysis (XRD) on a D 5000 X-Ray Diffractometer (Siemens, Karlsruhe, Germany), using CuKα radiation within a 2θ range of 5 – 40° at a scanning speed of 1.2° min^-1^. In addition, attenuated total reflectance with Fourier transform infrared spectroscopy (ATR-FTIR) analysis was performed using a Cary 630 Agilent Technologies FTIR-ATR (Agilent Technologies Inc., Santa Clara, CA, USA) spectrometer in the 400-4000 cm^-1^ wavenumber range.

Discs, before and after seven days of SBF immersion, were dehydrated, mounted on aluminium stubs, and coated with gold. Specimens were examined using a scanning electron microscope (Jeol JSM-IT300LV, JEOL USA Inc., USA) connected to an energy dispersive x-ray detector for elemental analysis with computer-controlled software, the Aztec EDS system (Oxford Instruments, Abingdon, UK). Micrographs of the material surface at 500x and 2000x magnifications were captured, and EDX quantitative chemical analyses were performed of BD, 1%nBG/BD, and 2%nBG/BD samples after seven days of SBF immersion at 2000x augmentation, representative areas for each material were analysed, and Ca/P ratio was calculated.

### Morphological and elemental analysis of the dentine-cement interface

The use of human extracted molars was approved by the Ethics Committee of the Faculty of Dentistry, University of Chile (PRI-ODO 14/011). Two-mm-thick dentine discs were obtained from human third molars via a water-cooled, low-speed diamond saw (Isomet, Buehler Ltd, Lake Bluff, IL, USA). The smear layer of the dentine discs was removed with 37% phosphoric acid for 20 seconds, and the discs were rinsed and dried with absorbent paper.

BD, 1%nBG/BD, and 2%nBG/BD were applied to one of the surfaces of the disc and allowed to fully set during incubation at 37°C and 100% humidity for 24 hours. An acid-etched dentin disc without cement application was used as control (CT). These specimens were then individually immersed in 50 mL of SBF, using polyethylene containers, for seven days at 37°C in a thermostatic bath**.**

Discs immersed in SBF were then dehydrated and fractured perpendicular to the dentine-cement interface. Specimens were mounted on aluminium stubs using carbon-coated, double-sided adhesive tape and then coated with gold. The discs were analysed using a scanning electron microscope with energy dispersive X-ray analysis (Jeol JSM-IT300LV, JEOL USA Inc). For morphological observations, dentine close to the dentine-cement interface was analysed. Representative micrographs of the interface were captured at 1000x and 2000x. EDX quantitative chemical analysis and element mapping was carried out with 2000x augmentation.

### Statistical analysis

The Ca/P molar ratio of EDX quantitative chemical analyses was calculated for BD, 1%nBG/BD, and 2%nBG/BD after seven days of SBF immersion (n=5) and statistical analyses were performed using SPSS 23.0 (SPSS Inc, Chicago, IL, USA) considering p<0.05 statistically significant. Normality was tested using Shapiro Wilk test, since data were not normally distributed for all groups; nonparametric Kruskal–Wallis and Mann–Whitney tests were performed for comparison of the groups.

## Results

### Characterisation and *in vitro* bioactivity of cements

**Figure 1:**
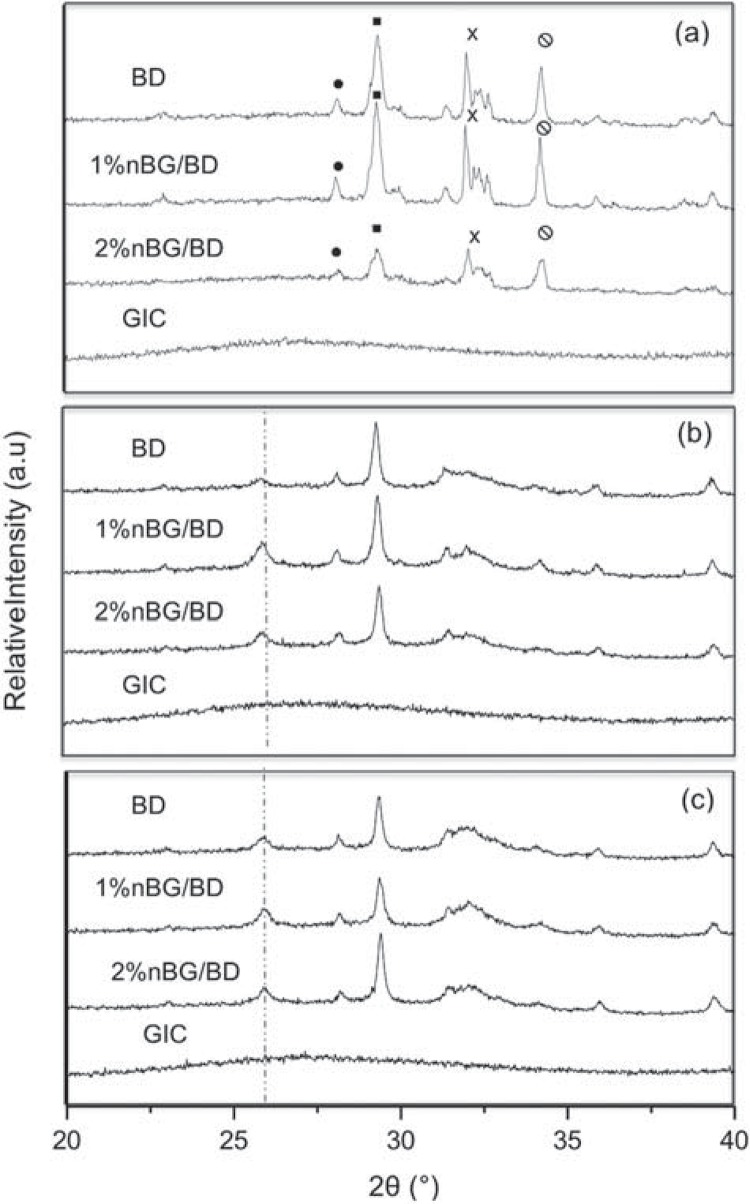
X-ray diffraction analysis of set cements (a) showing the main phases present [(circle: ZrO2, Baddeleyite; square: Calcite, CaCO3; X: Calcium Silicate, Ca3SiO5; Portlandite, Ca(OH)2] and after three days (b) and seven days (c) of immersion in SBF

The XRD patterns of the set cement materials are shown in Figure 1a. The BD diffractogram exhibits the characteristic peaks that correspond to the crystalline phases (baddeleyite, calcite, calcium silicate, and portlandite) that constitute the BD cement^1^. Nanocomposite samples showed similar XRD peaks, although they were less intense in the nanocomposite with greater nBG content (2%nBG/BD). The XRD patterns of the materials after immersion in SBF are shown in Figure 1b-c. After three days of incubation, nBG/BD nanocomposite diffractograms presented one of the most characteristic apatite reflections (ICDD® PDF No: 9-432) at 25.9° (Figure 1a). In the case of BD, the appearance of this apatite peak was only detected after seven days of incubation (Figure 1c). On the other hand, no XRD peaks were detected for the GIC before or after SBF incubation.

**Figure 2:**
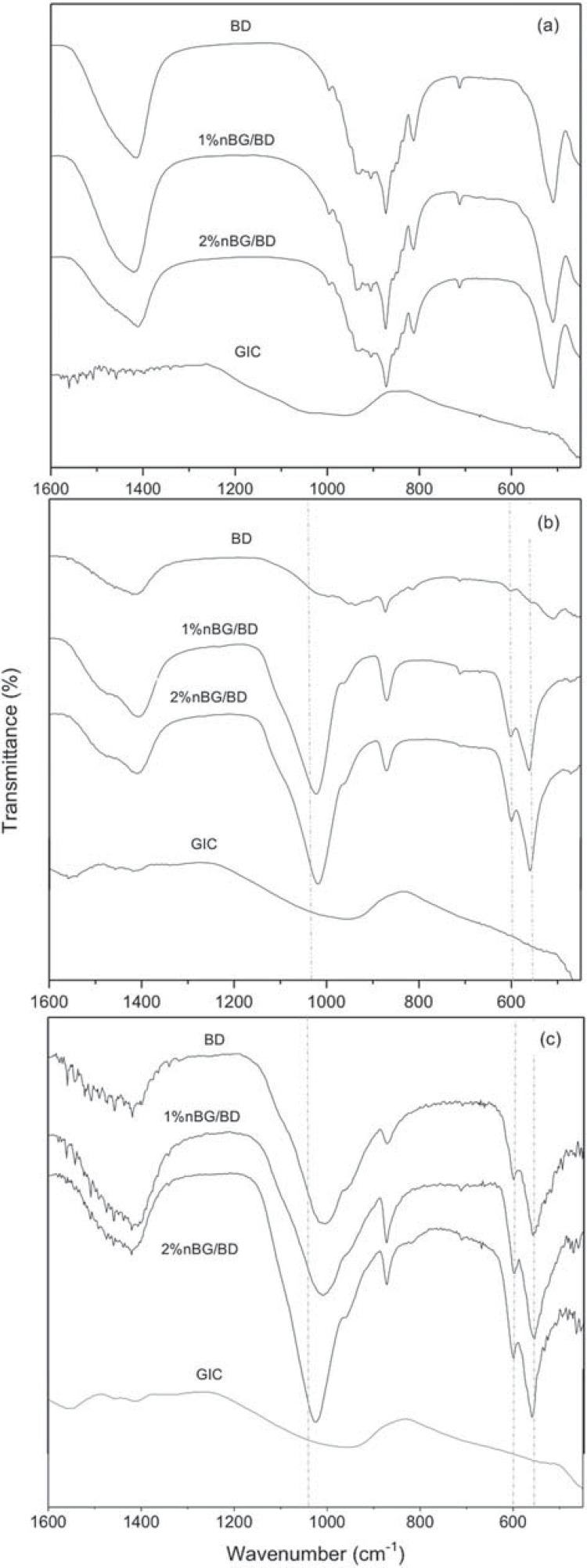
ATR-FTIR spectrum of set cements (a) and cements immersed in SBF for three days (b) and seven days (c)

The ATR-FTIR analysis of the cement materials in the 1600-450 cm^-1^ region is shown in Figure 2a. The BD and nBG/BD spectra presented bands at 520 cm^-1^ that corresponded to silica vibration modes and at 1400, 870, and 710 cm^-1^ that were atributed to the vibrations of carbonate bonds^29^ . GIC presents a wide and weak band around 1000 cm^-1^ caused by bridging (Si-O-Si) and non-bridging (terminal Si-O group) oxygen^1^. After three days of SBF immersion, the bands of the cement matrix components progressively disappeared in all of the materials (Figure 2b). The appearance of new bands at 560, 600, and 1040 cm^-1^ in the nBG/BF spectra is attributed to P-O vibrations of the apatite structure^17^; in contrast, these bands were hardly detected in the BD spectrum. After seven days of immersion (Figure 2c), apatite bands became more intense for all the cement materials, but particularly for the nBG/BD nanocomposites.

**Figure 3:**
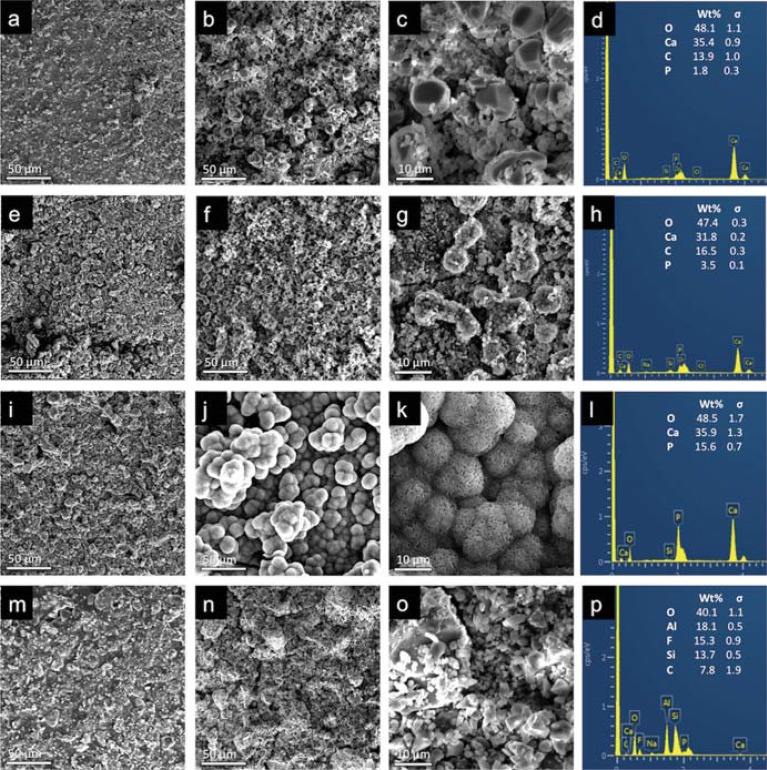
Scanning electron microscopy micrographs and EDX analysis of set cements before (a, e, I, m) and after seven days of SBF immersion (b-d, f-h, j-l, n-p) of BD (a-d), 1%nBG/BD (e-h), 2%nBG/BD (i-l), and GIC (m-p). Representative images at 500x (a-b, e-f, i-j, m-n) and 2000x (c, g, k, o) magnifications with EDX elemental analysis (d, h, l, p).

SEM/EDX images of samples before and after SBF soaking for seven days are presented in Figure 3. The presence of mineral deposits can be observed on the cement surfaces after immersion in SBF. The BD surface was not significantly modified after a period of SBF immersion. In contrast, the formation of a new mineral phase was clearly observed on the nBG/BD surfaces. Characteristic spherical apatite deposits covered the entire surface of the nanocomposites, and a denser and well-developed apatite layer was produced on 2%nBG/BD. EDX analysis showed that the mineral deposits that formed on BD were mainly composed of carbon, oxygen, calcium, and phosphorous, with increasing phosphorous content on the nBG-modified cements. The median (minimum-maximum) Ca/P molar ratio values of BD, 1%nBG/BD, and 2%nBG/BD surfaces were 15.32 (11.05 - 21.40), 4.08 (2.67 - 4.35), and 1.88 (1.70 - 3.16), and differences were statistically significant between all groups (p<0.05). On the other hand, no mineral deposit formation was detected on the GIC surface, and oxygen, aluminium, fluorine, silicon, and carbon appeared to be the main elemental components.

### *In vitro* bioactivity of the dentine-cement interface

**Figure 4:**
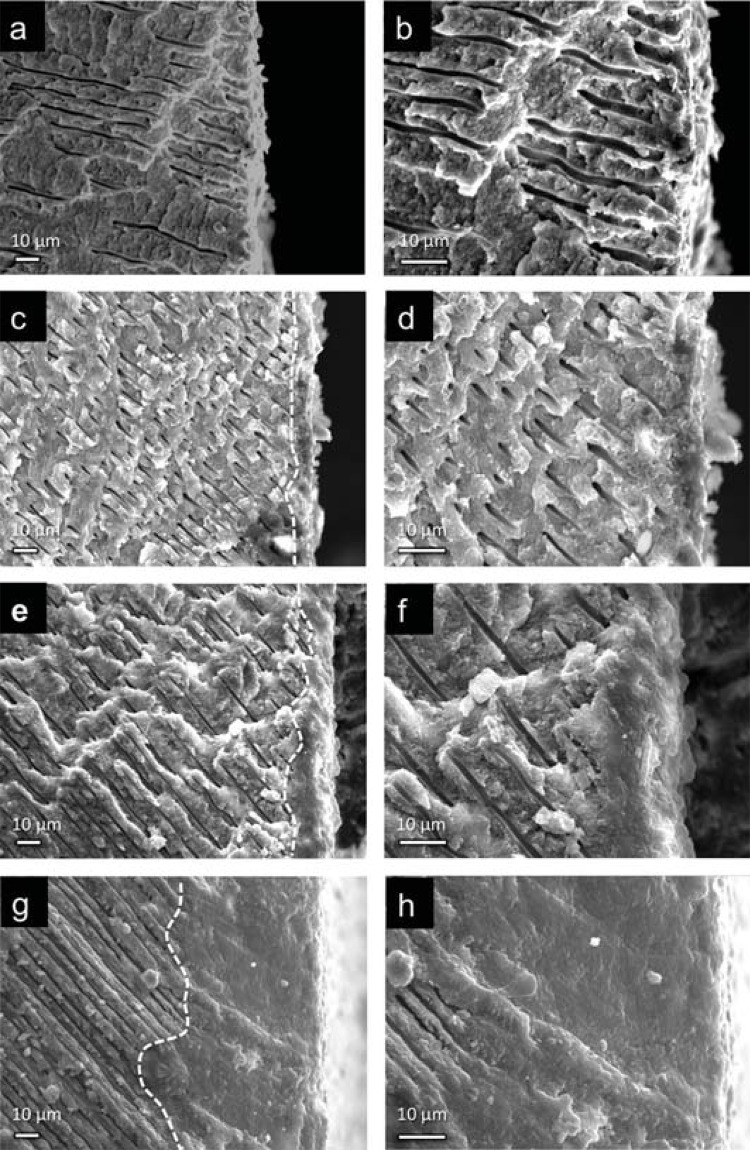
Scanning electron microscopy micrographs of dentine-material interface of CT (a-b), BD (c-d), 1%nBG/BD (e-f), and 2%nBG/BD (g-h) samples, in which material was on the left side before fracture. Dotted lines show the approximate position of the interfacial area

Cross-sectional SEM images of the cement-treated dentine interfaces after seven days of immersion in SBF are shown in Figure 4. The presence of an interfacial mineralised layer in the dentine treated with both BD and nBG/BD nanocomposites can be observed, which can be identified as an area of morphologically altered dentine where dentine tubules appeared blocked by the new mineral phase. This area was largely developed in the cement modified with nBGs; it appeared to be approximately four times thicker in the 2%nBG/BD nanocomposite than in BD. In contrast, in CT sample open and funnelled dentine tubules towards the surface are observed.

**Figure 5:**
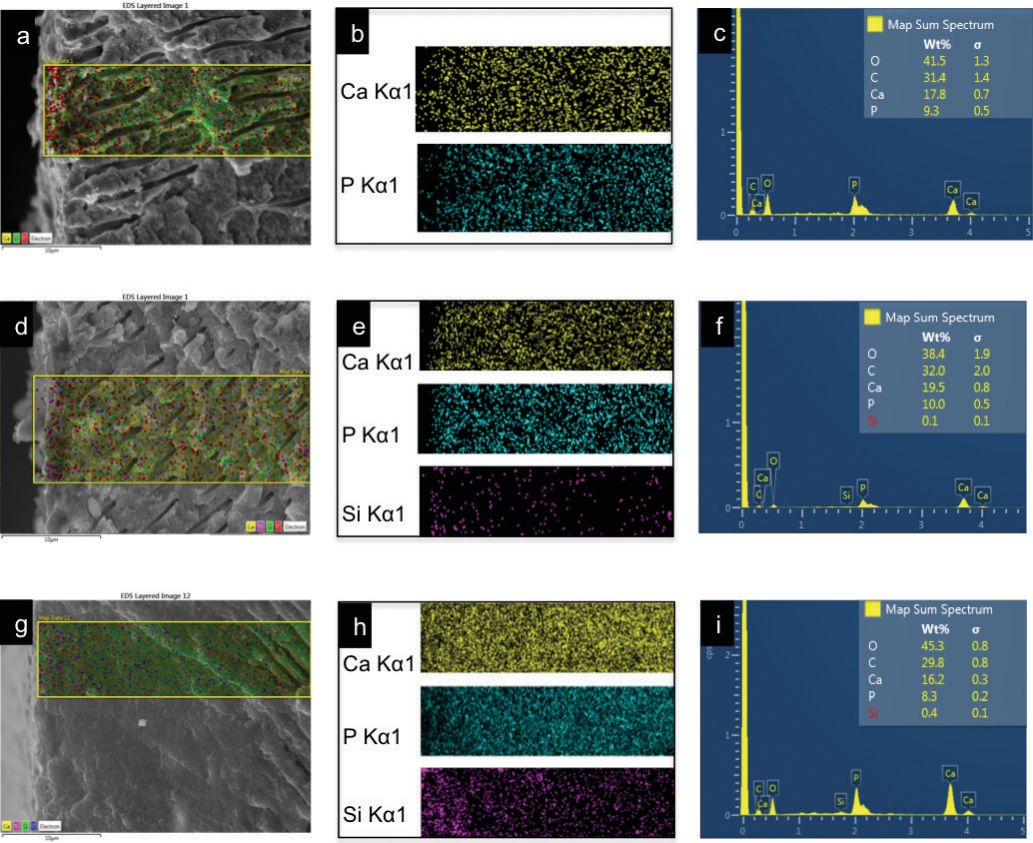
Micrographs and mapping images obtained by scanning electron microscopy (SEM) - energy dispersive (EDX) of the CT (a-c), BD-dentine (d-f), and 2%nBG/BD (g-i) interfacial areas. SEM micrographs at 2,000x with a selected area (a, d, g) for EDX mapping (b, e, h) and EDX bulk analysis (c, f, i)

Element mapping of BD and 2%nBG/BD samples (Figure 5) showed the distribution of calcium, phosphorous, and silica within the dentine treated with the cements. BD- and 2%nBG/BD-treated dentine presented equivalent calcium and phosphorous distributions, whereas the 2%nBG/BD- treated dentinal tissue had a higher silicon density, which decreased with dentinal depth.

## Discussion

This study demonstrated the feasibility of preparing bioactive cement nanocomposites, based on the incorporation of nBGs into the BD matrix. The assessment of nBG/BD nanocomposite bioactivity demonstrated accelerated apatite-phase formation, compared with unmodified BD. XRD analysis showed earlier apatite crystallisation (after three days), as judged by the appearance of one of the most characteristic apatite reflections at 25.90°, corresponding to the (002) plane of the apatite crystal structure (ICDD® PDF No: 9-432). ATR-FTIR similarly confirmed the earlier presence of apatite deposits on the nanocomposites by the appearance of characteristic PO_4_ vibrations of the crystalline apatite structure^17^. Consistently, SEM images showed surface precipitates with globular morphology on the nBD composites, which had the distinctive appearance of apatite deposits. Similar cauliflower-like clusters have been observed by others when nBGs were immersed in SBF^18^. In addition Ca/P value of nBG/BDs deposits was closer to the stoichiometric value of the hydroxyapatite structure (1.67)^25^ than BD deposits. Taken together, the combined structural, chemical, morphologic, and elemental analyses showed that the nanocomposites possess enhanced bioactivity, expressed by an accelerated formation of the crystalline apatite-phase on their surface when immersed in SBF, compared with BD.

The improved bioactivity exhibited by the nanocomposites can be directly attributed to the incorporation of nBGs into the BD matrix. BG is a bioactive material well known for its ability to form an apatite layer on its surface through solution-mediated dissolution of the glass structure^10^ ^,^ ^12^. Therefore, the results in this study suggest that when nBG was incorporated into BD and immersed in SBF, the bioactive ability of the nanoparticles was expressed. The dissolution of BG silicate network into ionic components forms a supersaturated solution, resulting in the nucleation of an amorphous calcium-phosphate layer, which is transformed into apatite^14^. This phenomenon was observed in the nanocomposites after shorter periods of immersion compared with BD, which is due to the rapid dissolution of the nBGs that can form an apatite layer in periods of 24 hours^3^. The bioactivity of BG has been also confirmed in *in vivo* and *in vitro* studies, demonstrating the formation of a bone-like apatite layer on its surface in the living body, and bonding to bone through this layer^10^ ^,^ ^12^. Other attempts to incorporate BG in restorative dental materials, such as resin adhesive^20^ and glass ionomer cements, have been reported^27^ ^,^ ^28^; however, to date there are no published studies assessing the effects of its incorporation into calcium-silicate-based materials. Moreover, BG particles have been incorporated into resin adhesive and glass ionomer cements by using traditional micrometre-size particles^20^ ^,^ ^27^ ^,^ ^28^, in contrast to the nanosized BG used in the current work.

nBGs present high surface area, with greater available surface for interaction with the physiological medium, which accelerates the ionic dissolution process of BG structure and, consequently, apatite formation and crystallisation^24^. This could explain why the incorporation of low levels of nBGs into the cement matrix (1% wt.) can improve the cement’s bioactivity, accelerating the formation of crystalline apatite.

In addition, there was a distinctive mineral-rich interfacial layer within the dentine in contact with BD and the nanocomposite cements, which was thicker and had greater Si uptake in the dentine treated with nBG-modified cement. This may indicate that the nanocomposites have more prominent mineralisation ability than unmodified BD. The ability of calcium silicate cements to mineralise dentine when immersed in fluids has been previously reported^8^ ^,^ ^21^. It is believed that the formation of this interfacial layer could be related to the good marginal seal of calcium silicate cements^8^, supported by reports in which immersion in fluids decreased the marginal leakage of apical plugs^19^ and increased push-out strengths^22^. For BD, this interfacial layer has also been named the “mineral infiltration zone”, which has been attributed to the dual effects of an alkaline caustic etching followed by mineral exchange^13^. This leads to the belief that the mineral deposits could reduce leakage by filling spaces along the interface and via interactions with dentine such as intrafibrilar apatite deposition^7^. The precise role of Si uptake remains unclear, but that silica uptake in dentine may increase its acid resistance and physical strength^8^.

In the present study, this interfacial layer was considerably thicker when BD was loaded with nBGs, possibly due to the additional effect of the bioactive ionic species produced by the nBG dissolution, which may diffuse along dentin tubules and induce the formation of apatite deposits. This new remineralisation layer could be formed by the penetration of the biomaterial into the open dentine tubules, and consequent transformation into an apatite phase by contact with the physiological medium. Silica is a key component of nBG and, following dissolution, could act as a nucleation site for the precipitation of dissolved calcium and phosphate ions to form hydroxyapatite^4^. This correlates well with the EDX mapping, where a higher Si content is observed in the mineral phase formed by nBG/BD. The presence of this morphologically different layer occluding previously open tubules suggested that the cement with nBG become lodge within the tubules, where apatite formation took place. Curtis, et al.^4^ (2010) have reported similar effect by brushing micro and nano-BG on dentine with exposed tubules. It was found that when nano-BG is applied, a rod-like apatite structure is formed within the tubules^4^, whereas only a surface layer apatite onto the tubule opening was detected with micro-BG. Therefore, the use of BG with nanometric dimensions strongly favours the BG diffusion into the tubules and its consequent transformation into apatite phase. In addition, demineralised dentine can be faster remineralised by nBGs than with micron-sized BG as consequence of the substantially higher rate of dissolution of nBGs^3^ ^,^ ^24^.

It would be interesting to study the long term stability of this interfacial layer in conditions that mimic the dynamics of its possible clinical applications. When BG has been added to other carriers, such as toothpastes, it has been demonstrated that the dentin tubule occlusion layer formed is resistant to acid challenge^5^, and reduces significantly dentine permeability under simulated oral environment^30^. In addition, it has been suggested that the apatite rods formed into dentine tubules after brushing with nBGs slurries would have excellent retention^4^. This is based on the observation that the continuous occluding apatite rods are tightly bonded to the dentine tubules, therefore the mechanical retention of these rods may be ensured as the angling and contours of the dentin tubule will avoid dislodgement of this interface^4^. Nevertheless, the study of the stability of the mineralised layer observed when nBG/BD was applied onto dentine would be of interest.

The outstanding ability of BD cement modified with nBGs to accelerate the formation of dentine-like crystalline apatite inside of dentine tubules could have favourable clinical consequences. nBG-modified cement could generate a strongly mineralised seal when moisture control is difficult or could remineralise dentinal tissue in restorative therapies.

## Conclusions

The incorporation of nBGs into BD enhances BD’s *in vitro* bioactive properties, accelerating the formation of a crystalline apatite layer on its surface after a short period of immersion in SBF and greatly enhances the formation of a mineral-rich interfacial layer when in contact with dentine.

## References

[1] Camilleri J (2013). Investigation of Biodentine as dentine replacement material. J Dent.

[2] Cao WP, Hench LL (1996). Bioactive materials. Ceram Int.

[3] Covarrubias C, Arroyo F, Balanda C, Neira M, Von Marttens A, Caviedes P (2015). The effect of the nanoscale structure of nanobioceramics on their in vitro bioactivity and cell differentiation properties. J Nanomater.

[4] Curtis AR, West NX, Su B (2010). Synthesis of nanobioglass and formation of apatite rods to occlude exposed dentine tubules and eliminate hypersensitivity. Acta Biomater.

[5] Farooq I, Moheet IA, AlShaimi E (2015). In vitro dentin tubule occlusion and remineralization competence of various toothpastes. Arch Oral Biol.

[6] Gjorgievska ES, Nicholson JW, Apostolska SM, Coleman NJ, Booth SE, Slipper IJ (2013). Interfacial properties of three different bioactive dentine substitutes. Microsc Microanal.

[7] Han L, Okiji T (2013). Bioactivity evaluation of three calcium silicate-based endodontic materials. Int Endod J.

[8] Han L, Okiji T (2011). Uptake of calcium and silicon released from calcium silicate-based endodontic materials into root canal dentine. Int Endod J.

[9] Hashem D, Mannocci F, Patel S, Manoharan A, Brown JE, Watson TF (2015). Clinical and radiographic assessment of the efficacy of calcium silicate indirect pulp capping: a randomized controlled clinical trial. J Dent Res.

[10] Hench LL (2006). The story of Bioglass. J Mater Sci Mater Med.

[11] Hilton TJ (2009). Keys to clinical success with pulp capping: a review of the literature. Oper Dent.

[12] Jones JR (2013). Review of bioactive glass: from Hench to hybrids. Acta Biomater.

[13] Kim JR, Nosrat A, Fouad AF (2015). Interfacial characteristics of Biodentine and MTA with dentine in simulated body fluid. J Dent.

[14] Kokubo T, Kim HM, Kawashita M, Nakamura T (2004). Bioactive metals: preparation and properties. J Mater Sci Mater Med.

[15] Kokubo T, Kushitani H, Sakka S, Kitsugi T, Yamamuro T (1990). Solutions able to reproduce in vivo surface-structure changes in bioactive glass-ceramic A-W. J Biomed Mater Res.

[16] Koubi G, Colon P, Franquin JC, Hartmann A, Richard G, Faure MO (2013). Clinical evaluation of the performance and safety of a new dentine substitute, Biodentine, in the restoration of posterior teeth: a prospective study. Clin Oral Investig.

[17] Lin Q, Li Y, Lan X, Lu C, Chen Y, Xu Z (2009). The apatite formation ability of CaF2 doping tricalcium silicates in simulated body fluid. Biomed Mater.

[18] Luz GM, Mano JF (2011). Preparation and characterization of bioactive glass nanoparticles prepared by sol-gel for biomedical applications. Nanotechnology.

[19] Martin RL, Monticelli F, Brackett WW, Loushine RJ, Rockman RA, Ferrari M (2007). Sealing properties of mineral trioxide aggregate orthograde apical plugs and root fillings in an in vitro apexification model. J Endod.

[20] Osorio R, Yamauti M, Sauro S, Watson TF, Toledano M (2012). Experimental resin cements containing bioactive fillers reduce matrix metalloproteinase-mediated dentin collagen degradation. J Endod.

[21] Reyes-Carmona JF, Felippe MS, Felippe WT (2009). Biomineralization ability and interaction of mineral trioxide aggregate and white portland cement with dentin in a phosphate-containing fluid. J Endod.

[22] Reyes-Carmona JF, Felippe MS, Felippe WT (2010). The biomineralization ability of mineral trioxide aggregate and Portland cement on dentin enhances the push-out strength. J Endod.

[23] Valenzuela F, Covarrubias C, Martinez C, Smith P, Diaz-Dosque M, Yazdani-Pedram M (2012). Preparation and bioactive properties of novel bone-repair bionanocomposites based on hydroxyapatite and bioactive glass nanoparticles. J Biomed Mater Res B Appl Biomater.

[24] Vollenweider M, Brunner TJ, Knecht S, Grass RN, Zehnder M, Imfeld T (2007). Remineralization of human dentin using ultrafine bioactive glass particles. Acta Biomater.

[25] Wang HB, Lee JK, Moursi A, Lannutti JJ (2003). Ca/P ratio effects on the degradation of hydroxyapatite in vitro. J Biomed Mater Res A.

[26] Watson TF, Atmeh AR, Sajini S, Cook RJ, Festy F (2014). Present and future of glass-ionomers and calcium-silicate cements as bioactive materials in dentistry: biophotonics-based interfacial analyses in health and disease. Dent Mater.

[27] Yli-Urpo H, Lassila LV, Narhi T, Vallittu PK (2005). Compressive strength and surface characterization of glass ionomer cements modified by particles of bioactive glass. Dent Mater.

[28] Yli-Urpo H, Vallittu PK, Narhi TO, Forsback AP, Vakiparta M (2004). Release of silica, calcium, phosphorus, and fluoride from glass ionomer cement containing bioactive glass. J Biomater Appl.

[29] Ylmen R, Jaglid U, Steenari BM, Panas I (2009). Early hydration and setting of Portland cement monitored by IR, SEM and Vicat techniques. Cem Concr Res.

[30] Zhong Y, Liu J, Li X, Yin W, He T, Hu D (2015). Effect of a novel bioactive glass-ceramic on dentinal tubule occlusion: an in vitro study. Aust Dent J.

