# ERRATUM

**DOI:** 10.1590/1678-7757-2017er001

**Published:** 2017

Due to a publishing error the article “Enhanced bioactive properties of BiodentineTMmodified with bioactive glass nanoparticles”, published at Journal of Applied Oral Science 25(2):177-85 was printed with the following error:

Page 177

Affiliation:

Eduardo FERNANDEZ^1^ should be Eduardo FERNANDEZ^1,4^

^1^Universidad de Chile, Facultad de Odontología, Departamento de Odontología Restauradora, Santiago, Chile

^2^Universidade Estadual Paulista - UNESP, Faculdade de Odontologia, Departmento de Odontologia Restauradora, Araraquara, Brazil.

^3^Universidad de Chile, Facultad de Odontología, Instituto de Investigación en Ciencias Odontológicas, Laboratorio de Nanobiomateriales, Santiago, Chile.

^4^Universidad Autónoma de Chile, Instituto de Ciencias Biomédicas, Santiago, Chile.

